# Influence of Viscosity on Variously Scaled Batch Cooling Crystallization from Aqueous Erythritol, Glucose, Xylitol, and Xylose Solutions

**DOI:** 10.1021/acs.cgd.3c01136

**Published:** 2024-03-21

**Authors:** Anna Zaykovskaya, Bernadeth Amano, Marjatta Louhi-Kultanen

**Affiliations:** Department of Chemical and Metallurgical Engineering, School of Chemical Engineering, Aalto University, Espoo 02150, Finland

## Abstract

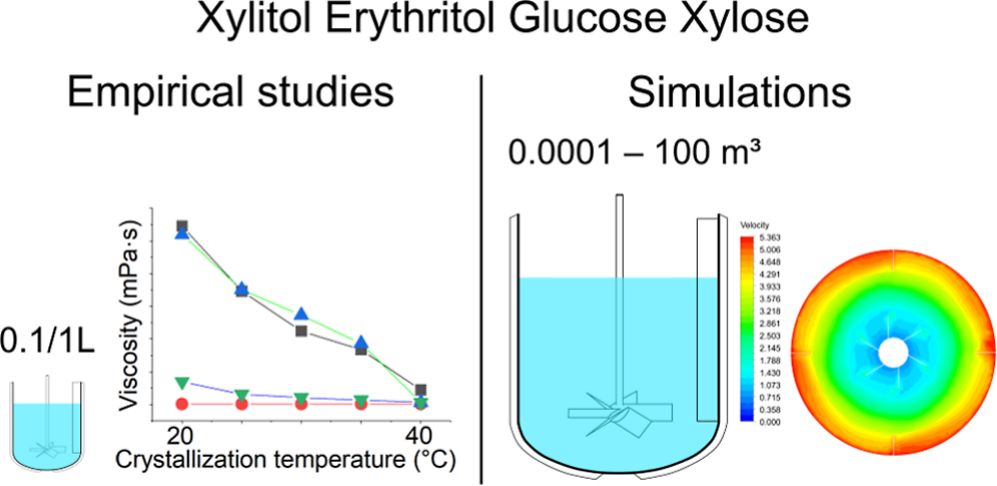

This study presents a comprehensive comparison of the batch cooling crystallization performance of aqueous solutions containing sugars and sugar alcohols, namely, erythritol, glucose, xylitol, and xylose. Erythritol and xylitol are commonly used alternative sweeteners to replace sucrose. They can be obtained by fermentation-based bioprocesses, where glucose and xylose are typical raw materials. These model compounds were selected based on their differing rheological nature: saturated erythritol solution has a viscosity lower than 3 mPa·s, whereas xylitol has the highest viscosity: greater than 90 mPa·s in the studied temperature range. Viscosities and densities of saturated solutions as well as apparent viscosities of crystal-mother liquor suspensions were measured. The purpose was to evaluate their crystallization behavior within a specific temperature range from 40 to 20 °C and batch time of 2 h, with the aim of understanding the influence of viscosity on the process more comprehensively. The comparison within the selected compound systems was carried out in terms of the physical properties of the mother liquor and the crystalline product. In addition to empirical laboratory-scale (0.1 and 1 L) studies, larger-scale simulations (1 and 100 m^3^) were performed with the experimental data obtained on average particle size, density, and viscosity for mother liquor and crystal-mother liquor suspensions. Mixing characteristics, such as the dissipation energy, mass transfer coefficient, energy of collisions, and micromixing time, were calculated with VisiMix software when using a single or dual impeller mixer. Furthermore, the scaling up of erythritol, xylitol, glucose, and xylose batch cooling crystallization from 40 to 20 °C based on the scaling-up rule of constant tip speed and energy of dissipation was done with VisiMix to obtain overall data on mixing conditions with crystallizers of 1 and 100 m^3^ in volume. Furthermore, ANSYS CFD software was used to determine the strain rates close to the impeller tip and velocity profiles on various crystallizer scales.

## Abstract 2

A thorough analysis of batch cooling crystallization for erythritol, glucose, xylitol, and xylose at 20–40 °C. Parameters studied: solution viscosities, densities, and apparent viscosities of crystal-mother liquor suspensions. Scale-up experiments with different impeller configurations, micromixing time, and energy dissipation. CFD modeling indicated the increasing role of viscous forces in the flow profile with scale, vital for process optimization. Viscosity is crucial for product quality and process optimization in scale-up.

## Introduction

Batch cooling crystallization is a well-established technique employed in various industries for the production of high-purity crystalline materials. In recent years, there has been increasing interest in the use of sugars and sugar alcohols as alternative sweeteners in numerous food and pharmaceutical applications. Among these alternatives, erythritol, xylitol, and xylose have attracted considerable attention due to their low calorie content, improved health benefits such as prevention of tooth decay, and suitability for individuals with diabetes. Glucose is one of the most common admixtures in industrial solutions, so it was investigated in the present study as well. However, a comprehensive comparison of their batch cooling crystallization performance, particularly in a specific temperature range, remains scarce in the literature.

The crystallization behavior of sugars and sugar alcohols in batch cooling processes has been investigated widely. Tyopkova et al.^1^ reported that a lower cooling rate increased the crystal size and changed the crystal morphology of erythritol. Gabas and Laguérie^2^ investigated the crystallization of xylose in batch cooling and antisolvent crystallization processes with ethanol addition, and determined the appropriate cooling rate and feeding rate of ethanol; they obtained the mass and crystal size distribution by Malvern analysis and population balance model of the produced d-xylose. In terms of viscosity measurements with the saturated mother liquors in our previous study,^3^ we investigated the effect of viscosity on the batch crystallization of xylitol by cooling, evaporative, and antisolvent crystallization. The results showed that the presence of ethanol enhanced the nucleation of xylitol, in addition to its effect of reducing dynamic viscosity. In addition, according to the focused beam reflectance measurement results of cooling crystallization obtained, the final total count rates per obtained crystal mass were higher at lower temperatures, which indicates that nucleation rates are higher in the studied temperature range.

The influence of viscosity on crystallization has been investigated with other compound systems as well. We examined the densities and viscosities of aqueous l-ascorbic acid solutions containing erythritol, xylitol, and mannitol in l-ascorbic acid aqueous solutions and highlighted the importance of viscosity in industrial areas concerning mass transfer, heat transfer, and fluid flow. In addition, Song et al.^5^ reported that the morphology of fullerene crystals can be controlled through kinetic overgrowth, which is influenced by the viscosity of the crystallization solution. This viscosity is manipulated by changing the temperature, allowing the formation of fullerene C70 crystals in distinct shapes such as concave cubes and octopods. The study emphasizes the role of solvent viscosity in crystal growth, suggesting that controlling the diffusion rate of organic molecules can effectively guide the morphology of organic crystals, a method that has been less explored compared to inorganic crystals. Similar to the findings of Jiang et al.,^4^ our previous research on galactose crystallization from a highly viscous industrial side stream also demonstrates a clear distinction in the morphological properties of crystallizing material. This study on crystallization conditions particularly underlines the influence of viscosity due to the presence of admixtures in high concentrations and the role of the selected temperature range on the obtained crystal size and purity of α-d-galactose.^6^ Ediger et al.^7^ investigated the relationship between the kinetic coefficient for crystal growth and the shear viscosity of supercooled melts with both organic and inorganic materials. To predict isothermal growth velocity of a crystal into its undercooled melt, the authors introduced an empirical expression including shear viscosity with an exponent that systematically varies depending on the fragility of the liquid. The fragility of the liquid is a measure showing how rapidly the viscosity of a liquid increases as it cools toward the transition temperature.

Despite these individual studies, a direct comparison of the batch cooling crystallization performances of erythritol, glucose, xylitol, and xylose within a specific temperature range is lacking in the literature. Understanding the similarities and differences in their crystallization behavior and the role of viscosity in these processes is crucial for optimizing the production of high-quality crystals by using these alternative sweeteners.

Industrial crystallization is commonly carried out in baffled stirred tanks.^8^ Depending on the rheological nature of the crystal-mother liquor suspension (CRY-ML suspension) and initial crystal-free mother liquor (ML), the dispersion of crystals in stirred tanks and the flow patterns of fluid and particles can vary significantly. Several factors can affect the quality of solid–liquid mixing, including tank geometry, impeller geometry, mixing intensity, baffles, density, and rheological properties.^9^ Central to this investigation are the key parameters, such as solution viscosities, densities, and the apparent viscosities of CRY-ML suspensions. As highlighted in the literature, these factors are fundamental determinants of product quality and efficiency in crystallization processes.^10,11^ The role of viscosity, in particular, is emphasized as a crucial factor for product quality and process optimization in scale-up, aligning with recent findings that underscore the increasing importance of viscous forces in flow profiles as the scale of operation increases.^12^ For instance, high viscosity can reduce the mass and heat transfer efficiency and further reduce the crystal growth kinetics. Thus, both the viscosity and rotation speed of the mixer have significant effects on the mass transfer coefficient values.^3,9^

The basic concept of VisiMix simulations is to model the flow of fluids and particles in a mixing reactor via computational fluid dynamics (CFD) and other mathematical models.^13^ VisiMix software was used in scale-up simulations to obtain the values of the average energy dissipation, crystal collision energy, and mass transfer coefficient for crystal and mother liquor suspensions in laminar and turbulent flow regions for single and dual impeller mixers. This is vital since impeller design and configuration significantly influence micromixing times and energy dissipation, both of which are pivotal in determining the particle size distribution (PSD), purity, and morphology.^11^ The complexity of scaling up crystallization processes is notably addressed through a quality by design approach, emphasizing the need for a structured and predictive method to manage these complexities.^10^ The required physical properties were the measured results obtained on a laboratory scale with the studied compounds.

In addition, it is possible to make process up-scaling simulations of the cooling crystallization process with VisiMix. Scaling up is a complex process, and the operational conditions employed in laboratory-scale crystallization may not apply to large crystallizers. Adjustment is required to compensate the differences in the crystallizer geometry, crystallizer configuration, and hydrodynamic and mixing conditions.^14^ Through careful process design, it is possible to scale up the cooling crystallization process while maintaining the quality and efficiency of the process, in terms of mass transfer and PSD of the final products.

The present work focused on up-scaling studies on the crystallization of erythritol, xylitol, glucose, and xylose using batch cooling from 40 to 20 °C in terms of constant tip speed or energy of dissipation. Furthermore, the empirical data for simulations were obtained in 2 h batch cooling crystallization in a temperature range from 40 to 20 °C. Besides VisiMix simulations, ANSYS using CFD modeling was carried out with the aim of investigating the influence of viscosity on fluid flow uniformity on various scales of cooling crystallization. Thus, incorporating advanced analytical techniques, including CFD and VisiMix modeling, and utilizing the accurate physical property data of the solutions, this paper aims to bridge the gap between small-scale experiments and industrial-scale operations.

## Experimental Section

### Empirical Laboratory-Scale Studies

#### Viscosity Measurements

A Brookfield DV-E viscometer was used to measure the viscosities of erythritol, xylitol, D-(+)-glucose, and D-(+)-xylose solutions at the selected shear rates and temperatures. The operating principle of the DV-E is to rotate a spindle (immersed in the test fluid) through a calibrated spring. The viscous drag of the fluid against the spindle is measured by the spring deflection, which is measured with a rotary transducer that provides a torque signal. The measurement range of DV-E (in milliPascal seconds) is determined by the rotational speed of the spindle (0.1–100 rpm), the size and shape of the spindle, the container in which the spindle is rotating, and the full-scale torque of the calibrated spring. In a typical experiment, the viscosity of a 16 mL sample was measured by using a Brookfield DV-E viscometer equipped with an appropriate spindle. Maintaining the temperature of the sample between 20 and 40 °C with a Lauda thermostat connected to the viscometer was crucial due to the impact of temperature on viscosity. The spindle was immersed to the specific depth in the sample, and the viscometer was allowed to stabilize for 30 min before the viscosity reading was recorded. Repeated measurements were carried out for the accuracy assessment, and the average of these readings was used as the final result. After each measurement, the spindle and chamber were thoroughly cleaned to prevent cross-contamination. All relevant details, including sample identity, spindle and speed settings, temperature, and viscosity readings, along with any anomalies during the measurement process, were meticulously recorded. The allowable error for the viscosity measurement is ±1% of full-scale range.

#### Liquid Density

The density of erythritol, xylitol, D-(+)-glucose, and D-(+)-xylose solutions saturated at 20 and 40 °C was determined using an Anton Paar DMA 5000 M density meter, where well-mixed and free of air bubbles solutions were fed. Adjusting the solution temperature to its equilibrium temperature was maintained using the integrated system of the density meter. During the filling process, care was taken to avoid introducing air bubbles into the density meter. Calibration was conducted using a standard reference material, followed by the sample measurement, which involved analysis of the oscillation frequency of a U-tube filled with the sample solution. To ensure accuracy and reproducibility, three measurements were taken and averaged. After the measurements were completed, thorough cleaning and regular maintenance of the density meter were performed to maintain measuring precision. Finally, all relevant details of the measurements, including sample identities, temperatures, and obtained density values, were meticulously recorded. The density measurements were performed to determine the quantities of chemicals required for the used reactor volumes, and they were needed in VisiMix and CFD simulations as well.

#### Solubility Measurements

A Dionex 3000 ICS high-performance anion exchange chromatography (HPAEC) system for quantitative carbohydrate analysis was used to determine the xylose solubility in water. Literature data were used, although only xylose solubility was investigated in the present work. Xylose samples were prepared at 20 and 40 °C and kept for 24 h with an excess xylose crystal to study solubility. Crystal-free samples for HPAEC were obtained by using a preheated syringe equipped with a microfilter. The solubility value was verified by repeating the measurements twice. It should be mentioned that data for xylose solubility at 20^15^ and 25 °C^16^ was as reported in the literature.

#### Particle Size Distribution Measurements

A Malvern Mastersizer 2000 instrument was used to analyze the PSD of the crystal sample materials. The device can be applied for particles in the size range 0.5–2000 μm. The samples were analyzed with the wet method, dispersing the sample in ethanol and then passing it through the laser diffraction instrument. By conducting the experiment five times, the consistency and reliability of the results were improved, as averaging the measurements can help mitigate the impact of any anomalies or outliers. If a particular measurement is identified as significantly deviant, it was excluded to avoid distorting the average result, ensuring a more accurate representation of the sample’s PSD.

#### Crystallization of Sugars and Sugar Alcohols

In the present work, erythritol (Sigma-Aldrich, ≥ 99%), xylitol (Sigma-Aldrich, ≥ 97.5%), D-(+)-glucose (Sigma-Aldrich, ≥ 99.5%), and D-(+)-xylose (Sigma-Aldrich, ≥ 99%) were crystallized by batch cooling crystallization from deionized Milli-Q water. A study was carried out using an EasyMax 402 stirred reactor workstation from Mettler Toledo. An up-pumping pitched four-blade stirrer with a diameter of 38 mm was used with a 100 mL reactor with a liquid volume of 80 mL. In addition, up-scaling experiments were performed in 1 L reactor with an up-pumping pitched six-blade stirrer with a diameter of 42 mm, a liquid volume of 800 mL, and mixing intensity of 700 rpm. The temperature probe was immersed inside the reactor.

Aqueous solutions of erythritol, xylitol, and glucose saturated at 40 °C were prepared for the studies on batch cooling crystallization based on the published solubility data,^17−19^ and in the case of xylose—based on our own measured data. A temperature range between 40 and 20 °C and a cooling rate of 10 K/h was used. The temperature probe was immersed inside the reactor. The estimated maximum errors are ±1.0 K for the jacket temperature and reactor temperature in the studied temperature ranges. As in the previous studies,^3^ a pitched-blade turbine with four blades with a diameter of 38 mm was used. The mixing intensity was 450 rpm, the diameter of the 100 mL glass reactor was 50 mm, and the solution content was 80 mL. Once the temperature reached 38.5 °C and the liquor became supersaturated, dry seed crystals were added through the reactor lid. The mass of added seeds was 1%^20^ of the theoretical crystal mass (*m*_th_), which was calculated from the theoretical solubility difference between 40 and 20 °C; the size of the seed crystals was about 50 μm. A similar seeding procedure^20^ was used in the majority of the batch cooling crystallization studies. The crystal size distribution of seed crystals was measured with a Malvern Mastersizer 2000 and the average size was 50 μm. In the case of xylitol crystallization, seed crystals were obtained by the rapid cooling of the xylitol solution and, for the other model compounds, by grinding the raw material.

#### Minimum Agitation Speed Determination for 100 mL Reactor

In 1958, Zwietering^21^ published a widely recognized article on solids suspension in which he adopted a rigid definition for calculating the just suspension speed (*N*_js_). According to Zwietering, this is the minimum stirring speed at which no solid particle remains stationary on the vessel base for more than 1 or 2 seconds. The accuracy of the measurement was claimed to be between 2 and 3%.

The Zwietering equation used for the minimum rotation speed determination is shown in eq 1
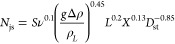
1where *N*_js_, just suspension speed, rps; *S*, geometrical constant, dimensionless; ν, kinematic viscosity (ν = μ/ρ), m^2^/s; *g*, acceleration due to gravity, m/s^2^; Δρ, density difference, kg/m^3^; ρ_*L*_, density of liquid, kg/m^3^; *L*, particle diameter, m; *X*, percentage mass ratio of solids to liquid in suspension, %; and *D*_st_*,* stirrer diameter, m.

In this eq 1, the viscosity of the liquid is represented by the kinematic viscosity term, ν. When the viscosity of the liquid is high, it indicates a higher resistance to flow, making it more difficult for the impeller to suspend and distribute the solid particles uniformly. As a result, a higher minimum rotation speed is required to overcome the increased viscosity and achieve suspension.

Incorporating the viscosity terms into the Zwietering equation provides a means of estimating the critical speed necessary for the suspension of solid particles in a liquid with a given viscosity.

### Calculations and Process Modeling

#### Scaling up

The first step in the scaling-up studies of cooling crystallization was to determine the size of the equipment. In the present work, we chose a stirred tank with an elliptical bottom with a total volume of 1 and 100 m^3^ for the scaling-up studies. A pitched blade dual impeller with six blades and diameters of 350 and 1625 mm was selected for the simulations. In addition, the reactors were equipped with four flat baffles for thorough/proper mixing of the CRY-ML suspensions. It should be mentioned that the mixing intensity must be carefully controlled to ensure that the agitation system does not create excessive shear forces to avoid undesired crystal breakage and attrition formation, which can increase the secondary nucleation rate and, thus, reduce the average crystal size. Therefore, mixing intensity is a critical parameter in the design and operation of crystallization equipment, having an impact on the heat and mass transfer coefficients in the system, which affects the crystal size distribution, crystal morphology, and overall crystallization efficiency. By appropriate adjustment of the mixing intensity, it is possible to optimize the crystallization process and produce high-quality crystalline products.

The mass transfer coefficient is another relevant parameter in the scaling up of cooling crystallization. With VisiMix, it is possible to fix this parameter and obtain the same mass transfer coefficient values based on up-scaling calculations. Therefore, in this case, a constant average value of turbulent dissipation in the tank is considered as the scale-up rule.

The optimal operational parameters of the cooling crystallization should be chosen to obtain a similar crystal size distribution to that in the laboratory-scale studies. Thus, we tried to reproduce the crystallization conditions used in the laboratory by using a temperature range between 40 and 20 °C and a cooling rate of 10 K/h. The measured crystal yields of the model compounds exceeded 90% of the theoretical yields therefore, we concluded that the cooling policy could be implemented for all four compounds.

#### Determination of Mass Transfer Coefficient

To compare different saturated solutions, we calculated their respective mass transfer coefficient values in a similar manner as in our previous studies.^3^ The mass transfer coefficient was determined using Levins and Glastonbury’s equation^22^ (see the Supporting Information) to compare it to the solid–liquid mass transfer coefficients obtained with VisiMix. The following expression for mass transfer coefficient between the particle and liquid was used in VisiMix calculations
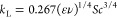
2where *k*_L_, mass transfer coefficient, m/s; ε, power input per unit mass of fluid, m^2^/s^3^; ν, kinematic viscosity (ν = μ/ρ), m^2^/s; and *Sc*, Schmidt number (ν/D), dimensionless.

#### Minimum Rotation Speed for Solid–Liquid Suspension

The minimum agitation speed for the 100 mL reactor was determined with the Zwietering equation, as shown in eq 1. In addition, *N*_js_ was determined with a VisiMix. Although VisiMix does not calculate the just suspension speed directly, it enables the user to determine this parameter easily.

#### Analysis of the Viscosity Effects on Flow Behavior with CFD Modeling

The effects of viscosity on flow behavior and scaling up were investigated by using CFD modeling. The strain rate, which relates the shear stress to viscosity, was determined. The velocity profiles for the different samples were also examined. It was assumed that these selected parameters would comprehensively illustrate the effects of viscosity on the sugar alcohol and sugar solutions studied, where the rheological nature differs.

ANSYS Fluent 23.2 was utilized to resolve the conservation equations for the mass and momentum in the fluid domain. The multiple reference frame (MRF) model was used, wherein a steady-state approximation is applied, and rotational speeds were assigned for each of the cell zones.

## Results and Discussion

### Results of Empirical Laboratory-Scale Studies

#### Viscosity Measurement Results

**Figure 1:**
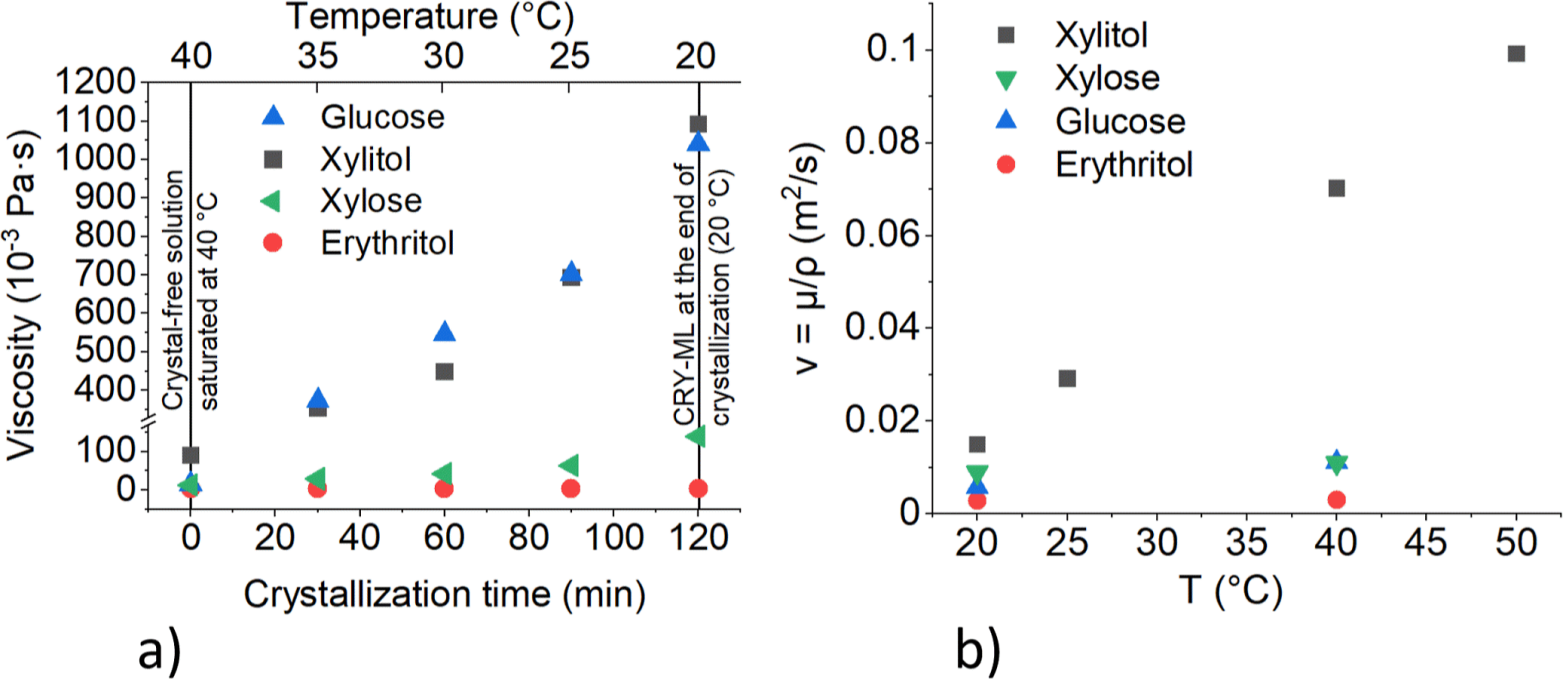
Viscosity measurement results: (a) apparent dynamic viscosity during crystallization. Crystals were present in the viscosity measurements at temperatures below 40 °C; (b) kinematic viscosity of saturated mother liquors.

**Figure 2:**
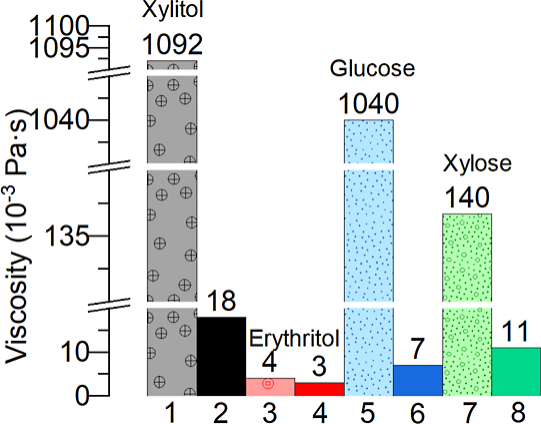
Comparison of viscosities of crystal-free mother liquor at 20 °C with apparent viscosities of CRY-ML suspensions at the end of crystallization. (1) Apparent xylitol suspension viscosity at 20 °C. (2) Viscosity of xylitol solution saturated at 20 °C. (3) Apparent erythritol suspension viscosity at 20 °C. (4) Viscosity of erythritol solution saturated at 20 °C. (5) Apparent glucose suspension viscosity at 20 °C. (6) Viscosity of glucose solution saturated at 20 °C. (7) Apparent xylose suspension viscosity at 20 °C. (8) Viscosity of xylose solution saturated at 20 °C.

The viscosity of the mother liquor is an important parameter that is often measured and monitored in industrial processes. The viscosity of the mother liquor also affects the efficiency of downstream processing such as filtration and drying. The apparent viscosity of a crystallizing suspension was measured in the temperature range used in the crystallization experiments. The results of the viscosity measurements are shown in Figure 1. In addition, Table A.1 demonstrates the measurement conditions and shear rates. The values in Figures 1 and 2 are average values of two measurements.

As can be seen from Figures 1 and 2, the viscosities were significantly changed by the presence of crystals. The only exception was erythritol. In the case of erythritol, the solubility and liquid viscosity at 20 °C are much smaller compared to other model compounds resulting in smaller apparent viscosity values and less noticeable difference between liquid and apparent viscosity values. In sugar and sugar alcohol solutions and solid–liquid suspensions, viscosity is an important property that impacts the efficiency of the process. Sugar and sugar alcohol solutions with high viscosities can be difficult to handle and may lead to clogging of pipelines and equipment. One of the factors that affect the viscosity of sugar solutions is the presence of crystals.

As expected, crystal-free mother liquors saturated at 20 °C had lower viscosities compared to those of crystal-ML suspensions at the end of crystallization. The crystal growth process is accompanied by a decrease in the sugar concentration in the mother liquor, leading to an increase in the viscosity. As more and more molecules crystallize, the sugar/sugar alcohol concentration in the mother liquor decreases and the viscosity continues to increase because of the lower temperature and the presence of crystals and thicker suspensions. The measured saturated mother liquor viscosity data were used in all the VisiMix and ANSYS simulations described below.

#### Liquid Density Measurement Results

**Figure 3:**
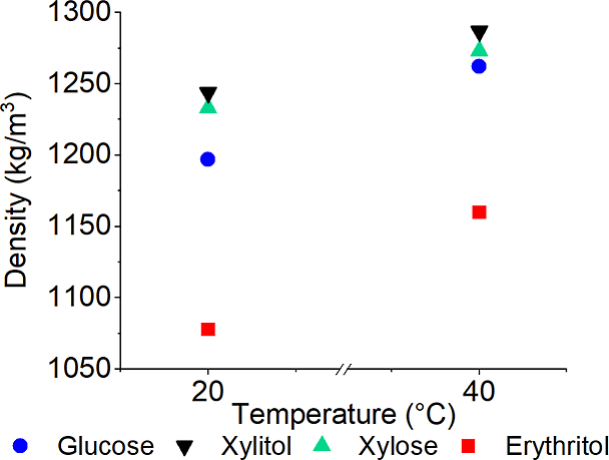
Density of solutions measured at their saturation temperatures at 20 and 40 °C.

Accurate measurement of the density and other related properties is essential for the optimization and scale-up of industrial processes. The results of the measurements of saturated solutions are shown in Figure 3. The results in Figure 3 are average values of three measurements.

#### Solubility of Model Compounds

**Figure 4:**
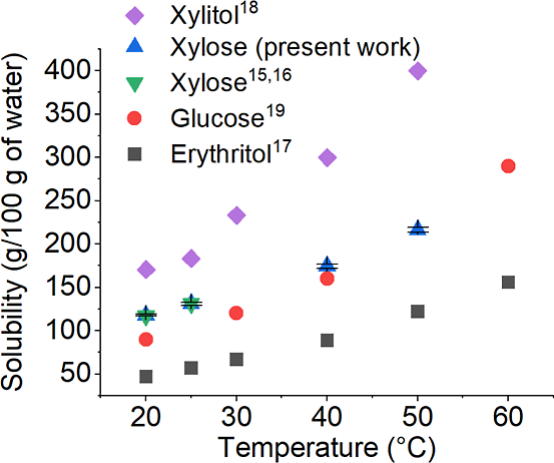
Solubility data reported in the literature and xylose solubility measured in the present work.

Solubility data are essential for the design and optimization of sugar crystallization processes. The solubility data of the model compounds in water was mainly found from the literature,^15−18,23^ but the solubility of xylose at 40 and 50 °C was measured in the present work. The measured xylose solubility data obtained at 20 and 25 °C were consistent with the literature data, as shown in Figure 4. The solubility of xylose in water was measured by HPAEC analysis, and the results are illustrated in Figure 4 and Table A.2.

Since xylose, xylitol, erythritol, and glucose are highly soluble in water and solubility changes with temperature, cooling crystallization is the most effective method to crystallize them. The results of xylose solubility measurements are average values of two measurements.

#### Crystallization Results

As mentioned above, we used the published solubility data^11−13^ for batch cooling crystallization, and partly our own measured data in the case of xylose.

Based on the obtained results, an *m*_obt_/*m*_th_ (obtained crystal mass/solubility based theoretical crystal mass × 100%) value of 99% was the highest in the case of xylose crystallization. The corresponding results for xylitol, erythritol, and glucose were 94, 91, and 98%, respectively. The crystallization results in both reactors used (100 mL and 1 L) showed very similar trends, and therefore crystallization was mainly performed in 100 mL reactor.

#### Particle Size Distribution Measurements

**Figure 5:**
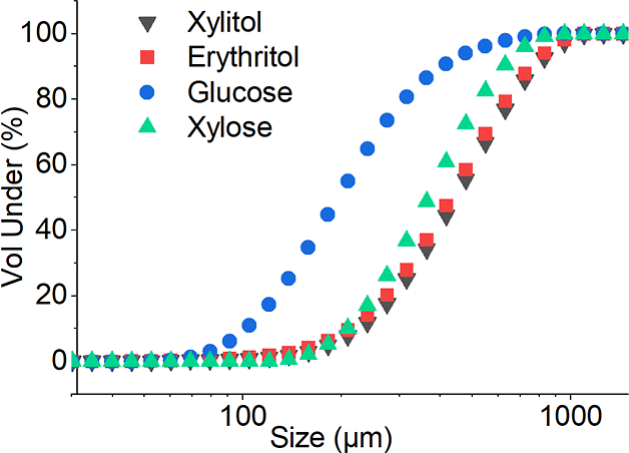
PSD measurement results.

PSD of the crystallized sugars and sugar alcohols was analyzed with a laser diffraction analyzer, and the results are shown in Figure 5.

To show the uniformity of the crystals, a cumulative curve was plotted. Based on the obtained data, glucose crystallization produced the smallest crystals, whereas xylitol crystallization produced the largest. The results of PSD measurements demonstrated in Figure 5 are average values of five measurements.

Simulation results were of 1 and 100 m^3^ crystallizers.

#### Reactors

**Figure 6:**
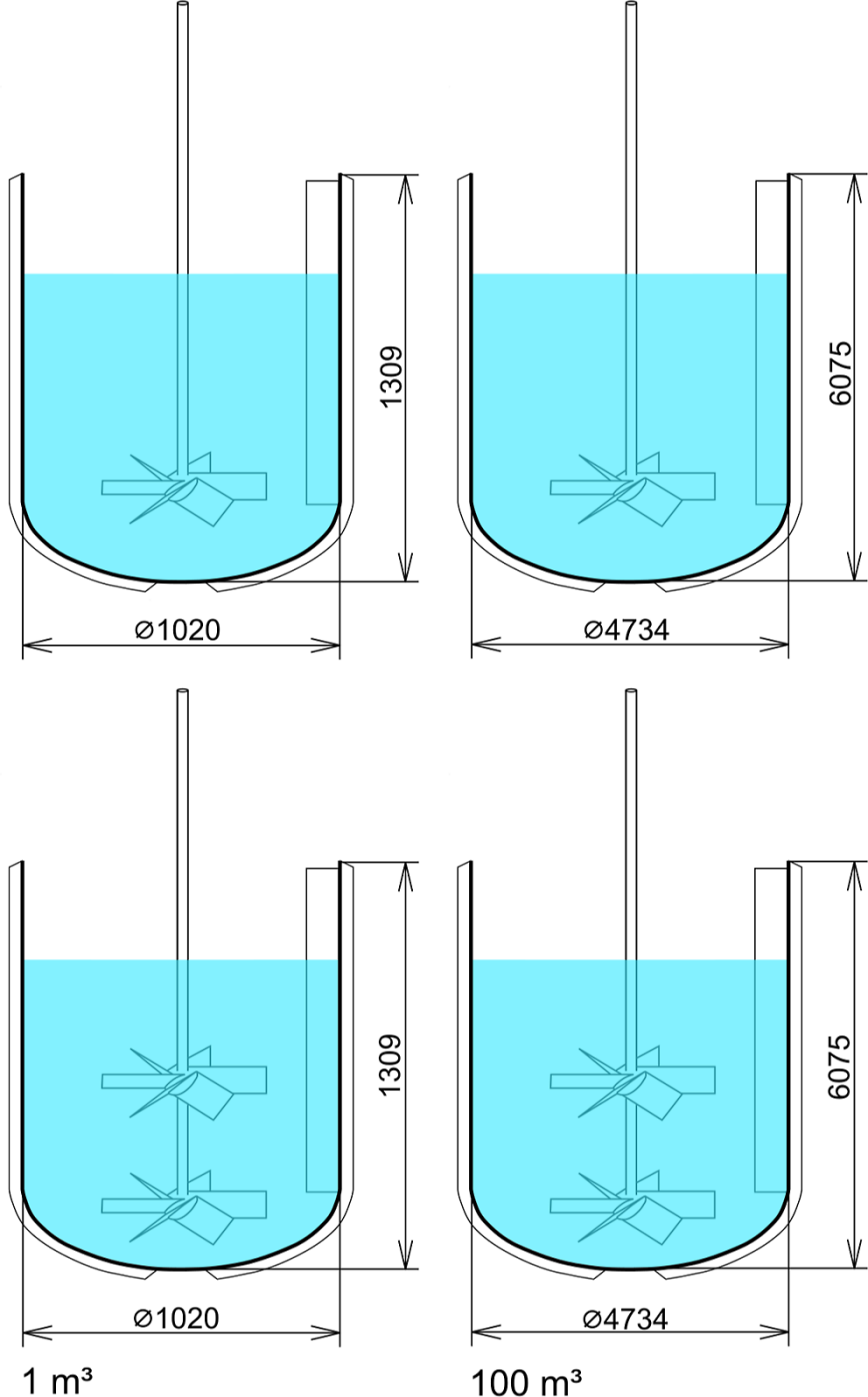
Large-scale crystallizers equipped with a single or dual impeller with six blades.

Figure 6 shows the dimensions of the chosen reactors. The detailed specifications of the reactors, impellers, and baffles are given in Table A.3. The mixer performance of single and dual impellers was compared.

#### Minimum Agitation Speed Determination Results

Agitation affects the mass transfer rate, crystal size distribution, crystal growth rate, nucleation, and further crystal size distribution. The appropriate agitation speed required for successful crystallization is dependent on several factors including the properties of the solution, the type of impeller used, and the desired crystal size and shape.

One of the primary factors to be determined is the minimum agitation speed required for crystals to be properly dispersed in the crystallizer. As discussed above, the viscosity of the solution is one of the critical properties that influences the minimum agitation speed, as well. Higher viscosity solutions require higher agitation speeds to achieve effective mixing and ensure uniform supersaturation throughout the solution.

The type of impeller used is another factor that determines the minimum agitation speed required for successful crystallization. The impeller design influences the mixing intensity and energy input required to achieve the desired supersaturation level.

The agitation speed affects the crystal growth rate, and higher agitation speeds can lead to smaller crystals with a narrower size distribution. Lower agitation speeds, on the other hand, can lead to larger crystals with a broader size distribution. The crystal shape is also influenced by the agitation speed, with higher agitation speeds leading to more spherical crystals and lower agitation speeds resulting in more irregularly shaped crystals, usually due to high supersaturation levels locally in poorly mixed regimes.

The Zwietering equation was used to calculate the minimum rotation speed to attain a better dispersed solid–liquid suspension in a stirred tank. The detailed data used for calculations are shown in Supporting Information, Table A.4. Tables 1 and 2 demonstrate the results of minimum agitation speeds that can be used in the crystallization process under the chosen conditions.

**Table 1:** Minimum Agitation Speed at 20 °C Calculated Using the Zwietering Equation for a Single Impeller

model compound	*N*_js_, rpm, 100 mL	tip speed, m/s, 100 mL	*N*_js_, rpm, 1 m^3^	tip speed, m/s, 1 m^3^	*N*_js_, rpm, 100 m^3^	tip speed, m/s, 100 m^3^
xylitol	528	1.05	79.98	1.46	22	1.84
glucose	507	1.01	76.84	1.41	21	1.77
xylose	406	0.81	61.55	1.13	17	1.42
erythritol	346	0.69	52.34	0.96	14	1.21

**Table 2:** Minimum Agitation Speed at 20 °C Calculated with VisiMix Software for a Single and a Dual Impeller

model compound	single impeller		dual impeller		single impeller		dual impeller	
	*N*_js_, rpm, 1 m^3^	tip speed, m/s, 1 m^3^	*N*_js_, rpm, 1 m^3^	tip speed, m/s, 1 m^3^	*N*_js_, rpm, 100 m^3^	tip speed, m/s, 100 m^3^	*N*_js_, rpm, 100 m^3^	tip speed, m/s, 100 m^3^
xylitol	85	1.57	87	1.59	23	1.96	19	1.62
glucose	72	1.31	66	1.21	23	1.96	18	1.53
xylose	67	1.23	63	1.14	21	1.79	17	1.45
erythritol	99	1.81	101	1.85	25	2.13	22	1.88

**Table 3:** Results of Scaling up Based on Impeller Tip Speed for Erythritol

temperature	40°C		20°C	
tank volume, m^3^	1	100	1	100
tank diameter, mm	1020	4734	1020	4734
rotation speed, rpm	100	21.6	100	21.6
**impeller tip speed,m/s**	**1.83**	**1.80**	**1.83**	**1.80**
energy dissipation–average value, W/kg single/dual impeller	0.105/0.189	0.0230/0.041	0.105/0.189	0.0230/0.041
maximum local energy dissipation rate, W/kg single/dual impeller	8.70/7.62	1.90/1.60	8.70/7.62	1.90/1.60
turbulent shear rate near the impeller blades, 1/s single/dual impeller	1730/1620	800/750	1520/1420	700/660
characteristic time of micromixing, s single/dual impeller	19.2/9.60	41/21	20.1/10.5	43/23
impeller Reynolds number	69,800	320,000	53,600	250,000

**Figure 7:**
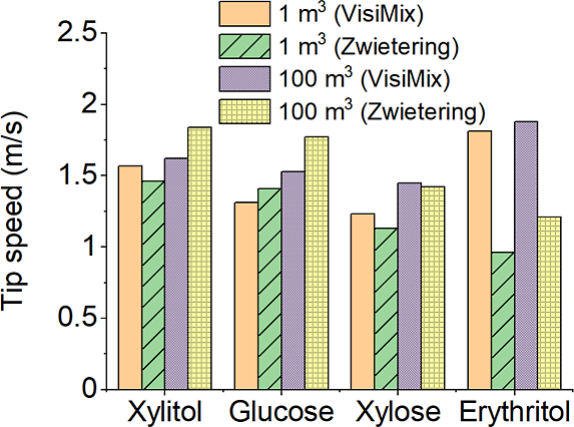
Comparison of minimum agitation speed at 20 °C calculated by using VisiMix software and the Zwietering equation for a single impeller.

Figure 7 shows a comparison of the minimum agitation speed at 20 °C calculated using VisiMix software and the Zwietering equation for a single impeller. Using the data we have gathered, we employed a tip speed of approximately 1.83 m/s in our subsequent simulations. This value was chosen to ensure that it is higher than or equal to the suspension speed in nearly all cases, facilitating a fair and comprehensive comparison of the simulation outcomes.

#### Scaling up

The most commonly used scaling-up rules are based on fixing the impeller tip speed, average/maximum energy dissipation rate, maximum shear rate, micromixing time, and impeller Reynolds number. In this work, the parameters to be reproduced and used as scale-up rules are the impeller tip speed (Table 3) or average energy dissipation (Table 4).

The appendix contains Tables A.4–A.9 with similar calculations for all of the studied compounds.

#### Energy Dissipation

**Figure 8:**
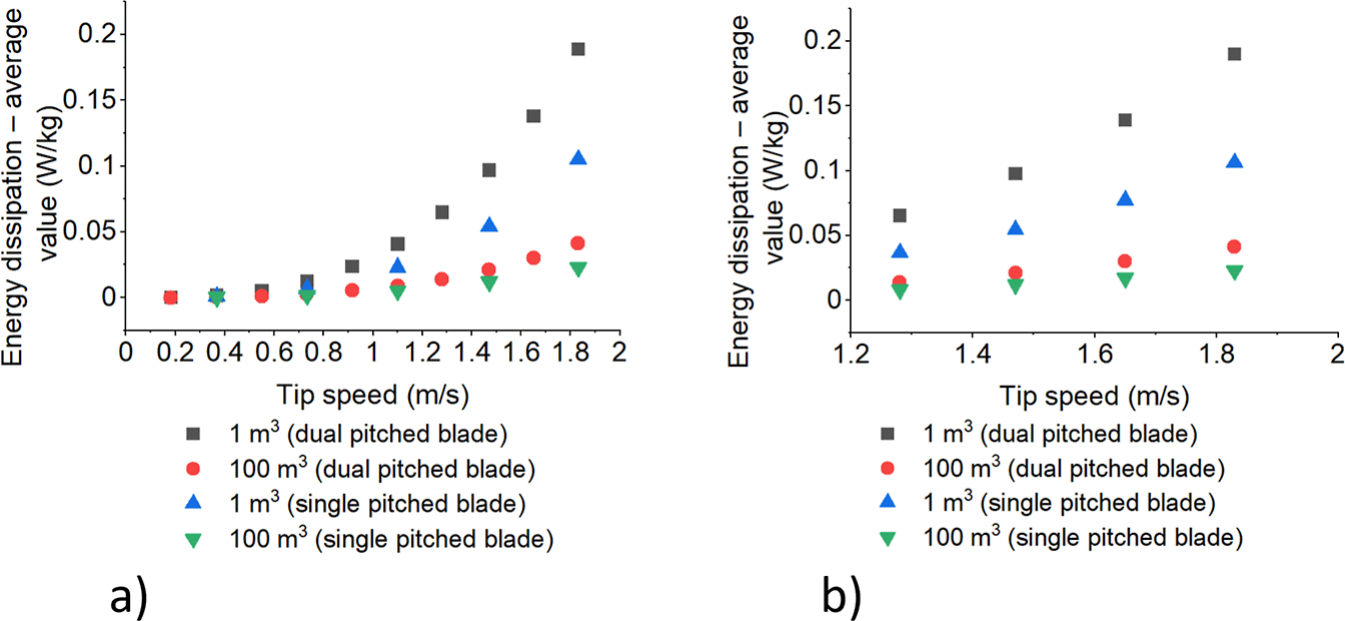
Average dissipation energy values for the compared mixing conditions single and dual stage pitched-blade mixer versus impeller tip speed on example of (a) erythritol and (b) xylitol at 40 °C.

The dissipation energy and impeller tip speed are two common parameters that have a significant impact on the crystallization process, determining the size and quality of the crystals formed. Figure 8 shows the dependence of average dissipation energy values on impeller tip speed for the compared mixing conditions (single- and dual stage pitched-blade mixer).

Dissipation energy is the energy that is lost due to internal friction or turbulence within a fluid. In the crystallization process, this energy is dissipated due to the movement of the impeller, which creates turbulence and shear forces within the solution. The amount of dissipation energy is proportional to the impeller tip speed. As the impeller tip speed increases, the turbulence and shear forces within the solution also increase, leading to greater energy dissipation.

The impeller mixing intensity is an essential parameter that affects the quality and size of the crystals formed. Extremely high mixing intensity used in thick suspensions can break the crystals due to secondary nucleation, as in our previous results with xylitol.^3^ The impeller speed also affects the uniformity of the supersaturation level in the crystallizer, which is a critical parameter that determines the crystal growth rate.

Supersaturation is the driving force behind crystal growth in the crystallization process. When the supersaturation level is high, the crystal growth rate can also be relatively high. The impeller speed affects the supersaturation level distribution. An appropriate impeller speed can lead to more efficient mass transfer, which can unify the supersaturation level and, therefore, the crystal growth rate. In cooling crystallization, it is favorable for the supersaturation to be quite uniform and at a moderate level in various locations of the crystallizer.

However, the relationship between the dissipation energy and impeller speed in the crystallization process is not straightforward. The optimal impeller speed and dissipation energy depend on various factors such as the solute concentration, solution viscosity, and impeller design. The design of the impeller is crucial because it determines the type and level of turbulence and shear forces within the solution.

#### Micromixing

**Figure 9:**
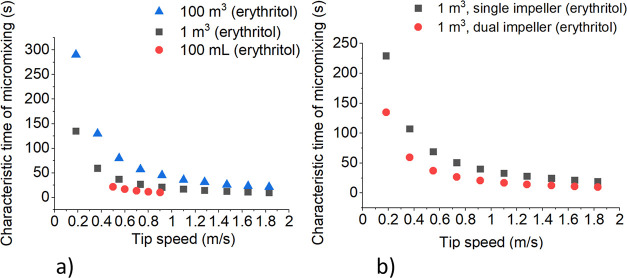
Characteristic time of micromixing vs tip speed of single impeller in erythritol crystallization at 40 °C: (a) comparison of the micromixing time in different reactor volumes and (b) comparison of the micromixing time with a single and a dual impeller.

Micromixing is a measure of the time required for the concentration of a species to become uniform throughout a given volume of fluid.^24^ It determines the efficiency of mixing and the rate at which crystallization can occur. The characteristic time of micromixing is influenced by the impeller speed. A higher impeller tip speed leads to a more intense mixing process and can result in faster micromixing. Figure 9 shows the relationship between the characteristic time of micromixing and the tip speed.

Figure 9 shows the relationship between the impeller speed and the characteristic micromixing time. The comparison of characteristic micromixing time in different reactor volumes can reveal how scale affects the mixing process. For instance, as can be seen from Figure 9 the characteristic micromixing time becomes independent of scale at high tip speeds. In addition, in larger volumes, achieving uniform mixing can be more challenging, potentially leading to variations in the micromixing efficiency. Moreover, the use of a dual impeller may lead to faster micromixing. These data can be crucial for optimizing erythritol crystallization processes. By understanding the relationship among impeller speed, characteristic micromixing time, and reactor volume, operational conditions can be adjusted to achieve desired crystal characteristics. Furthermore, characteristic micromixing time is one of the factors which can be taken into account in the design and scaling up of industrial crystallizers. Knowing how impeller speed affects the characteristic micromixing time across different volumes can guide the choice of reactor size and mixing equipment for efficient and cost-effective operations.

Moreover, additional VisiMix simulations and experiments with a 1 L crystallizer were carried out to investigate the influence of viscosity on mixing times of a pH increase with saturated xylitol solutions at 20 and 40 °C. In the experiments, the response curves were obtained by measuring pH over time after pulsewise addition of the acid sample. The obtained pH response curves and VisiMix characteristic time for mixing times are presented in Figure A.3.

**Figure 10:**
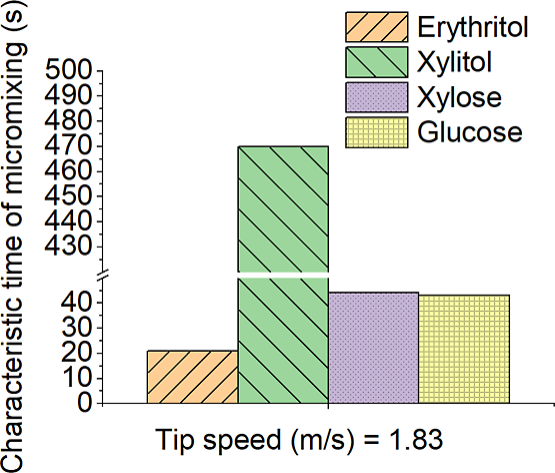
Comparison of characteristic time of micromixing of erythritol, xylitol, xylose, and glucose at 40 °C with a tip speed of 1.83 m/s and a dual impeller in a 100 m^3^ reactor.

Based on Figure 10, xylitol has the longest micromixing time, mainly due to the large average crystal size, as well as high viscosity. Glucose has a shorter micromixing time even though it is highly viscous, but on the other hand, the crystal sizes are smaller.

In addition to the impeller tip speed, other factors can also influence the characteristic time of micromixing. For example, the size and geometry of the mixing vessel can affect the flow patterns within the fluid and the efficiency of mixing. Similarly, the viscosity of the fluid can affect the rate at which species diffuse within the fluid and, therefore, can impact the characteristic time of micromixing.

In general, a higher impeller tip speed leads to a shorter characteristic time of micromixing, but at very high velocities, this relationship may begin to plateau and even reverse. As such, it is important to carefully consider the relationship between these two parameters when mixing processes are designed for a wide range of applications.

#### Maximum Energy of Collisions

This parameter characterizes^25^ the energy of collisions in the zone of maximum turbulence, predominantly concentrated near the impeller blades. In this region, where the fluid dynamics are most intense, the collisions between particles possess significant energy. The magnitude of this energy plays a pivotal role in determining the outcomes within the system.

**Figure 11:**
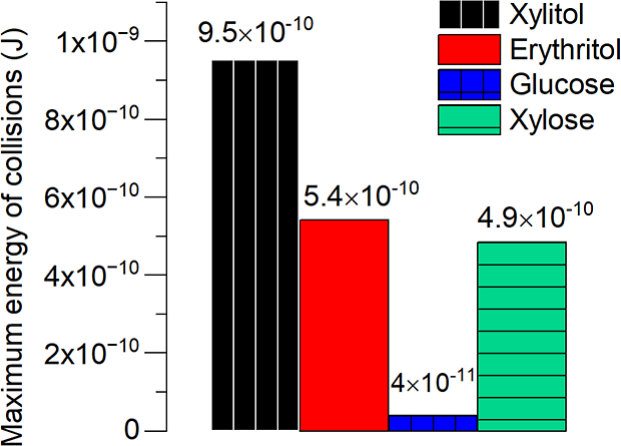
Maximum energy of collisions of xylitol, erythritol, glucose, and xylose at 20 °C in a 1 m^3^ reactor and 100 rpm.

By virtue of their influence, higher energy levels are directly correlated with increased rates of crystal breaking and secondary nucleation. The heightened intensity of collisions can induce fractures in the crystal lattice, leading to the formation of smaller crystal fragments. Additionally, elevated energy levels facilitate the occurrence of secondary nucleation, where new crystals are generated as a result of collision-induced disruptions. Figure 11 shows the maximum collision energy values for the materials studied.

The relationship between the crystal size and collision energy becomes evident when examining Figure 11 and the corresponding PSD measurements. It is apparent that, as the crystal size increases, so do the values associated with the energy of collisions.

Thus, understanding and monitoring the maximum energy of collisions are crucial for optimizing processes that involve turbulence and crystal formation. By carefully managing the energy of collisions within the zone of maximum turbulence, we can regulate crystal size, distribution, and overall product quality.

**Table 4:** Results of Scaling up Based on the Average Value of Energy Dissipation for Erythritol

temperature	40°C	20°C		
tank volume, m^3^	1	100	1	100
tank diameter, mm	1020	4734	1020	4734
rotation speed, rpm	100	36	100	36
impeller tip speed, m/s	1.83	3.10	1.83	3.10
**energydissipation–averagevalue,W/kg** single/dual impeller	**0.105/0.189**	**0.110/0.190**	**0.105/0.189**	**0.110/0.190**
maximum local energy dissipation rate, W/kg single/dual impeller	8.70/7.62	8.70/7.60	8.70/7.62	8.70/7.60
turbulent shear rate near the impeller blades, 1/s single/dual impeller	1730/1620	1700/1600	1520/1420	1500/1400
characteristic time of micromixing, s single/dual impeller	19.2/9.60	19.0/9.60	20.1/10.5	20/10
impeller Reynolds number	69,800	540,000	53,600	410,000

#### Mass Transfer

**Figure 12:**
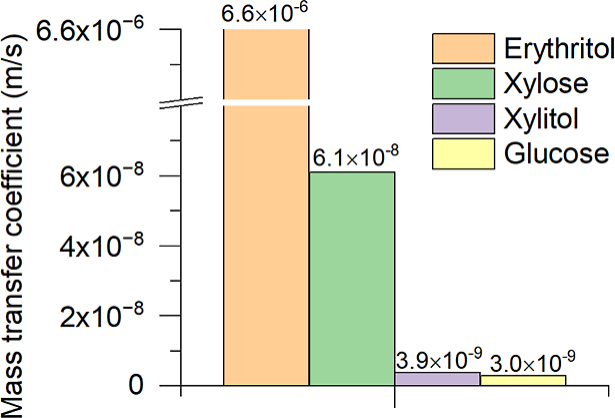
Mass transfer calculation results at 20 °C with a 100 m^3^ crystallizer, mixing intensity of 36 rpm, and single impeller calculated obtained with VisiMix.

Table 5 and Figure 12 show the solid–liquid mass transfer coefficients for the materials studied.

**Table 5:** Calculated Mass Transfer Coefficient Values Were Obtained with VisiMix

crystallizing material	crystallization conditions		
	20°C, 100 m^3^, 36 rpm		
	mass transfer coefficient, m/s	viscosity, 10^–3^ Pa·s	median size L_50_, μm
erythritol	6.6 × 10^–6^	4	423
glucose	3.0 × 10^–9^	1040	200
xylitol	3.9 × 10^–9^	1092	480
xylose	6.1 × 10^–8^	140	400

The mass transfer coefficient calculation results show that the higher viscosity of a xylitol CRY-ML reduces mass transfer. Erythritol with a CRY-ML viscosity of 4 × 10^–3^ Pa·s had the highest mass transfer coefficient of 6.6 × 10^–6^ m/s, whereas glucose had 1040 × 10^–3^ Pa·s and the lowest mass transfer coefficient of 3 × 10^–9^ m/s.

Mass transfer coefficients for saturated crystal-free solutions were calculated using Levins and Glastonbury’s equation^22^ for comparison with the solid–liquid mass transfer coefficients obtained with VisiMix (Figure 12). The detailed data used in the calculations of the xylitol mass transfer coefficient in a 100 m^3^ crystallizer as well as the expression reported by Levins and Glastonbury for calculating the mass transfer coefficient are given in Table A.11.

**Figure 13:**
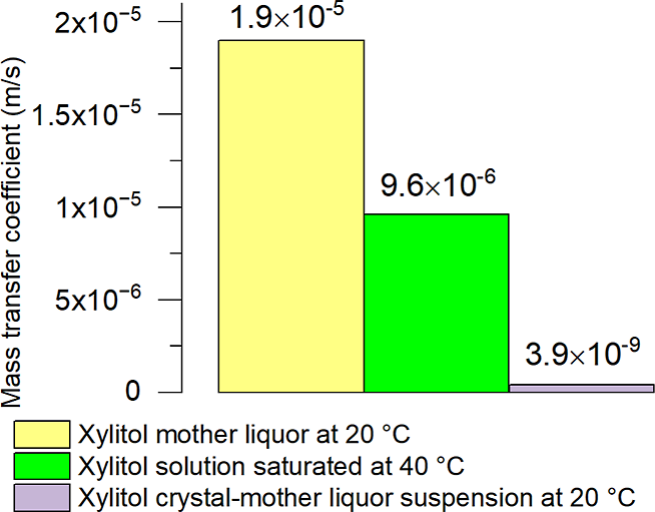
Mass transfer calculation results of xylitol obtained at 20 °C with a 100 m^3^ crystallizer, a mixing intensity of 36 rpm, and a single impeller. The mass transfer of xylitol mother liquor at 20 °C and xylitol solution saturated at 40 °C were calculated using the Levins and Glastonbury equation, whereas the mass transfer coefficient of the xylitol CRY-ML suspension at 20 °C was obtained with VisiMix.

Figure 13 shows a comparison of the mass transfer coefficients of crystal-free saturated xylitol solutions and CRY-ML suspensions.

The comparison of the mass transfer coefficients of crystal-free saturated xylitol solutions demonstrated the same trend—the mass transfer coefficient decreases with increasing viscosity.

#### CFD Modeling Results

In CFD modeling, a reactor setup was designed following the scales, as presented in Table A.3. Employing the MRF technique, two zones were created with an inner domain containing the impeller and an outer domain to represent the rest of the reactor volume. The geometry was scaled to 0.0001 m^3^ (A), 1 m^3^ (B), and 100 m^3^ (C) volumes. The domain is discretized, and the mesh quality was optimized by assessing the skewness and orthogonality of the cells. A skewness of 0.25 (excellent) and an orthogonal quality of 0.7 (0 is the worst and a value of 1 is the best)^26^ were achieved. Based on these results, the values were deemed to be within the acceptable range.

Isothermal and steady-state conditions were applied. The empirically obtained data described in the text above were also utilized. The single-phase sample solutions at 40 and 20 °C were used. Frame motion is assigned to the inner domain, with an impeller tip speed of 1.83 m/s used for all simulations. As we are uncertain about the flow characteristics, the shear stress transport k-omega model is used, as its formulation takes into account different flow behaviors. Convergence criteria are set to a residual target of 10^–6^.

Using this constant tip speed as a scale-up rule, the model was validated by assessing the average velocity profile throughout the domain.

**Figure 14:**
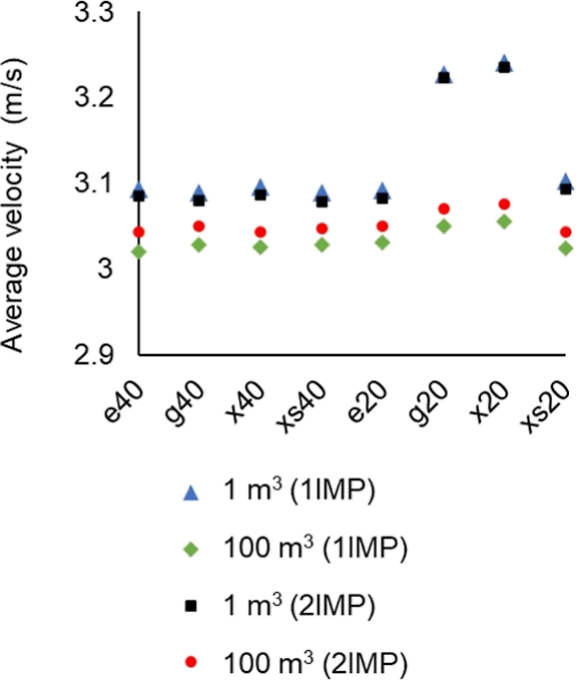
Average velocity at 1 and 100 m^3^ for single (1IMP) and dual (2IMP) impeller reactors. Erythritol, glucose, xylitol, and xylose at 40 °C (e40, g40, x40, and xs40) and 20 °C (e20, g20, x20, and xs20).

The results are presented in Figure 14.

Based on Figure 14, it can be observed that the average velocity remains in the same range and magnitude at different scales, with some fluctuations that could be attributed to the varying tank dimensions. Therefore, for the purpose of this study, with a constant tip speed and fairly/reasonably constant average velocity, the model can be deemed acceptable and used to assess the viscosity effects and velocity profiles of the samples in this study. A higher average velocity can be noted for glucose and xylitol (g20 and x20) for all cases. These two components have the highest viscosity out of all of the modeled solutions, which could reduce turbulence in the fluid flow compared to low viscosity solutions. It could also be that these more viscous solutions tend to move as clumps more than the others, thus leading to a higher average velocity. It is worth noting that the deviations in the average velocity of these solutions decrease as the scale increases.

#### Strain Rate

**Figure 15:**
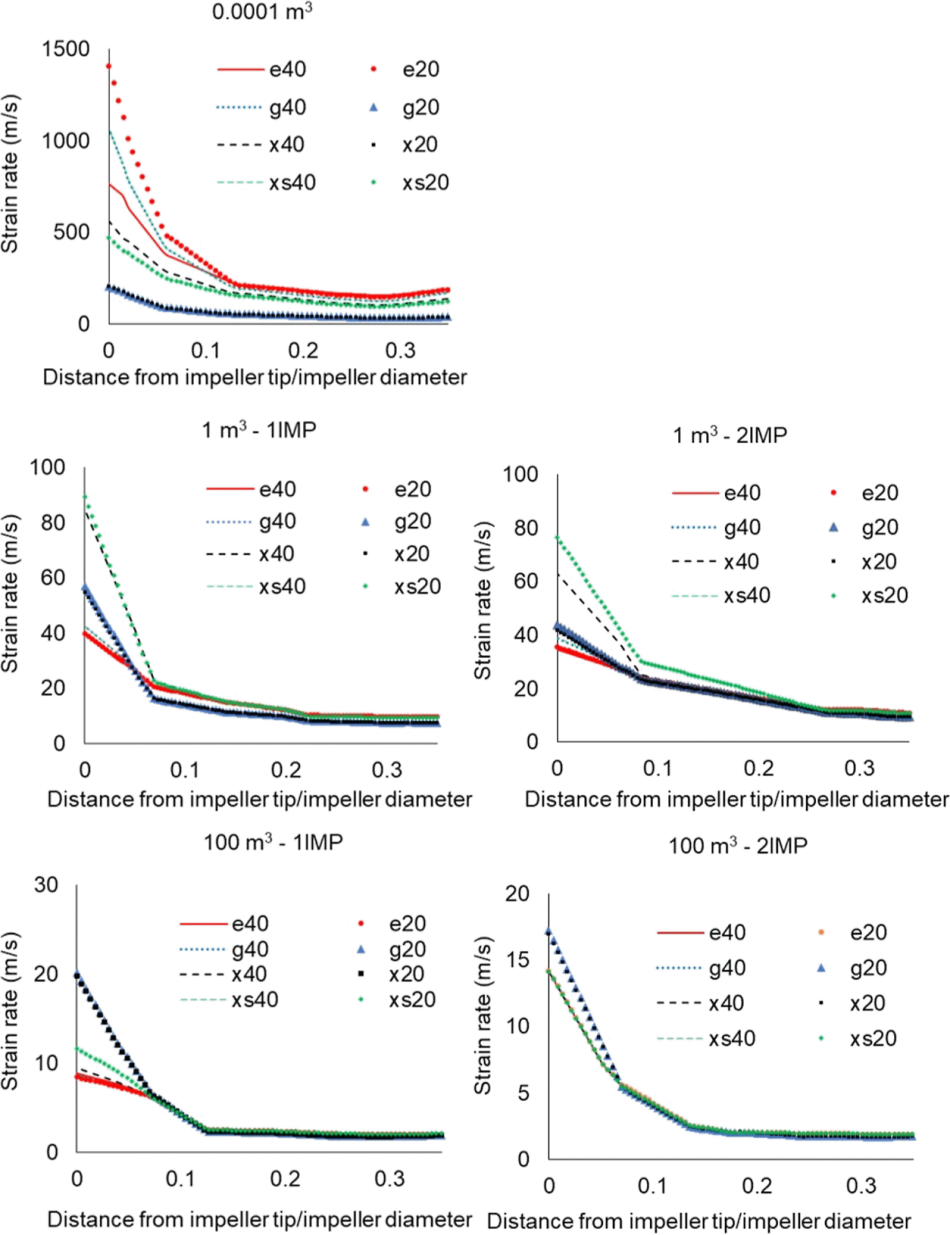
Strain rates at 0.0001, 1, and 100 m^3^ (1IMP-single, 2IMP-dual impeller). Erythritol, glucose, xylitol, and xylose at 40 °C (e40, g40, x40, and xs40) and 20 °C (e20, g20, x20, and xs20).

To compare the influence of viscosity on the fluid flow, the strain rates near the impeller tip region were calculated for each solution at three different scales. It was assumed that higher strain rate values would indicate a greater influence of friction force in high-viscosity solutions. A virtual probe was placed in a uniform location at the tip of the impeller blade to assess the strain rate values by using the different samples at various scales. Figure 15 presents the strain rates of the samples on different scales.

At the 0.0001 m^3^ scale, as shown in Figure 15, erythritol at 20 °C possesses the highest strain rate, with glucose and xylose at 20 °C having the lowest strain rate. At this scale, it can be observed that the less viscous sample is exposed to a higher strain rate.

In the 1 m^3^-scale single impeller case, xylitol at 40 °C and xylose at 20 °C have the highest strain rates, followed by glucose and xylitol at 20 °C, with the latter two having similar viscosities. For this case, erythritol has the lowest strain rates at both temperatures. The same could be observed in the dual impeller case except that there are higher strain rate differences between xylose at 20 °C and xylitol at 40 °C.

In the 100 m^3^ scale single impeller case, glucose and xylitol at 20 °C have the highest strain rates, followed by xylose at 20 °C, whereas the other samples have very similar values. The same could be observed in the dual impeller case, except that xylose at 20 °C now also has values similar to the others, while glucose and xylitol at 20 °C have the highest strain rates. For high-viscosity solutions and depending on rotational speed, turbulent forces dominate the flow profile at small scales. However, as the reactor scale increases, the flow is significantly affected by viscous forces.

**Figure 16:**
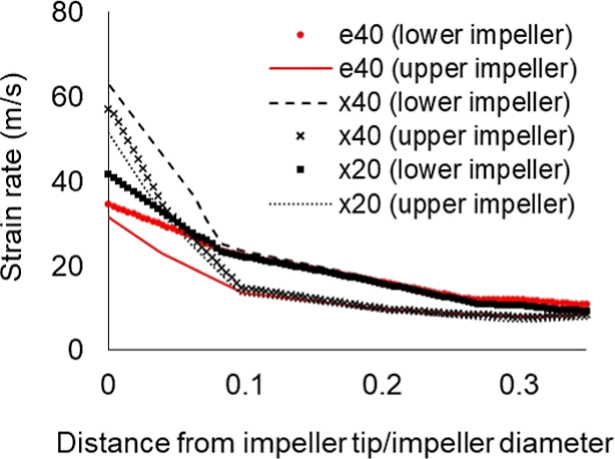
Strain rates of 1 m^3^ (2IMP-dual impeller blade) for erythritol and xylitol at 40 °C (e40 and x40) and 20 °C (x20).

The strain rates of xylitol and erythritol in a dual impeller reactor are plotted in Figure 16. It can be observed that, for less viscous solutions, the upper impeller has a lesser strain rate than the lower impeller, but for viscous solution (x20), it is the opposite. Moreover, while the impeller experiences high strain rates at a smaller scale, they decrease more as the scale increases. This is observed from the results of strain rates obtained, shown in Figure 15, where the values for the 0.1 L crystallizer differ greatly from the results of the 1 and 100 m^3^ crystallizers. For this case, higher strain rates are concentrated in the baffle instead, as presented in Figure A.1.

#### Velocity Profile

**Figure 17:**
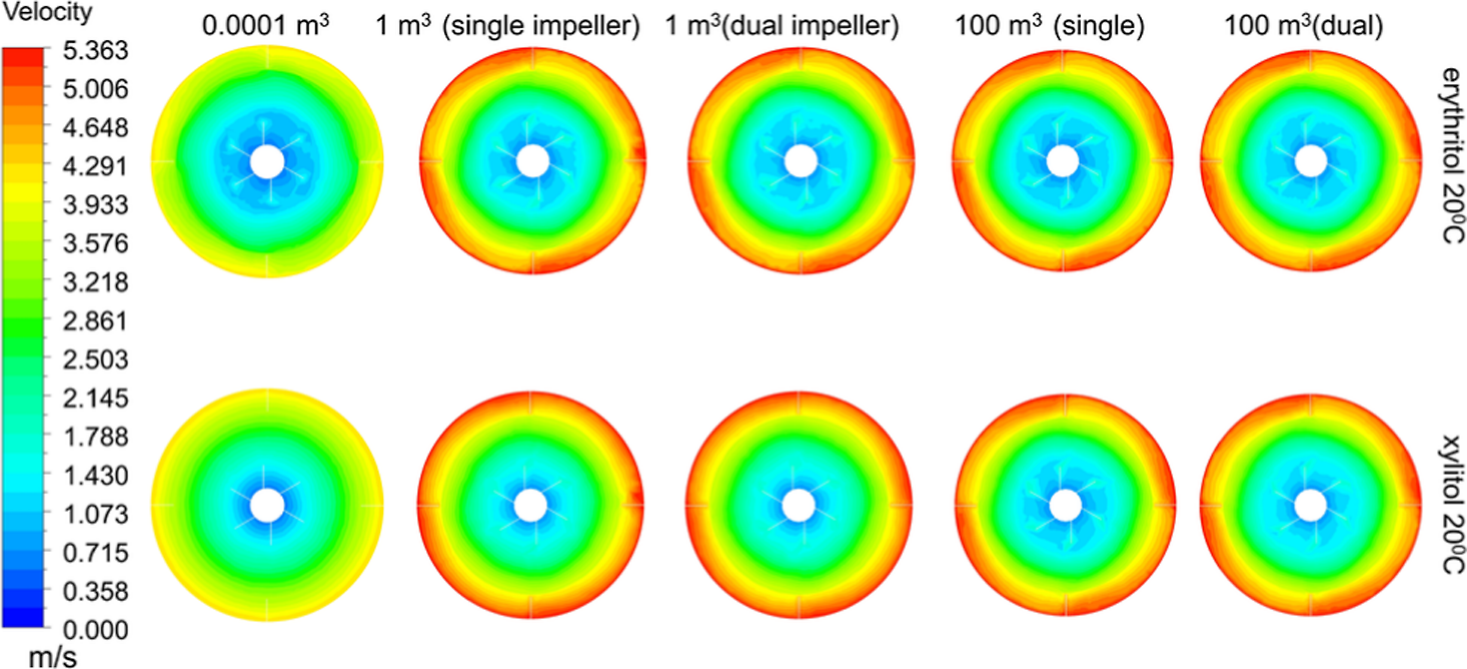
Velocity profile of xylitol and erythritol at 20 °C. From left to right: 0.0001, 1 m^3^ (single, dual impeller), and 100 m^3^ (single, dual impeller).

Figure 17 presents a comparison of the velocity profile for xylitol and erythritol at 20 °C. The profiles for all other samples are included in the Supporting Information.

The velocity profile at different scales is demonstrated for the highly viscous compound, xylitol, against erythritol, which has lower viscosity. It can be observed that erythritol retains a fairly similar profile throughout the scaling up. This comparably well-distributed flow could be attributed to the low viscosity of erythritol, leading to less friction in the fluid and allowing more movement, whereas xylitol appears to clump up on each scale, with the inner domain having the same velocity as the impeller tip.

## Conclusions

The present work presents a thorough analysis of batch cooling crystallization performance for solutions of selected sugars and sugar alcohols, specifically, erythritol, glucose, xylitol, and xylose. Various parameters were measured, including the viscosities and densities of saturated solutions, as well as the apparent viscosities of CRY-ML suspensions. The main objectives were to assess the crystallization behavior of these compounds within a specific temperature range (40 to 20 °C), understand the impact of viscosity on the process, and compare the physical properties and crystal products of the selected systems. The laboratory-scale crystallization results of the study revealed that the obtained crystal mass related to theoretical crystal mass (*m*_obt_/*m*_th_) was the highest for xylose crystallization, reaching 99%. In comparison, xylitol, erythritol, and glucose exhibited *m*_obt_/*m*_th_ values of 94, 91, and 98%, respectively. The PSD of the crystallized sugars and sugar alcohols was examined by using a laser diffraction analyzer. Analysis of the data revealed that glucose crystallization resulted in the formation of the smallest crystals, whereas xylitol crystallization yielded the largest. Furthermore, the apparent viscosity of the suspensions undergoing crystallization was measured within the temperature range employed in the experiments. The viscosities were found to be significantly influenced by the presence of crystals. As anticipated, the crystal-free mother liquors saturated at 20 °C exhibited viscosities lower than those of the suspensions containing crystals toward the end of the crystallization process.

The study encompassed both empirical laboratory-scale experiments with 0.1 and 1 L crystallizers and process-scale simulations with 1 and 100 m^3^ crystallizers. Using VisiMix software, several mixing characteristics such as the dissipation energy, tip speed, mass transfer coefficient, energy of collisions, and micromixing time were calculated. Thus, the study established a correlation between crystal size and the energy of collisions. It was observed that, as the crystal size increased, the corresponding collision energy values also increased. In addition, the results of the mass transfer coefficient calculations indicate that higher viscosities of the mother liquor hinder mass transfer. Among the compounds studied, erythritol, with a mother liquor viscosity of 4 × 10^–3^ Pa·s, exhibited the highest mass transfer coefficient of 6.6 × 10^–6^ m/s. In contrast, glucose, with a mother liquor viscosity of 1.04 Pa·s, demonstrated the lowest mass transfer coefficient of 3 × 10^–9^ m/s. Furthermore, the scaling up of batch cooling crystallization for erythritol, xylitol, glucose, and xylose, from 40 to 20 °C, was performed based on constant tip speed and energy of dissipation using VisiMix. In addition, the findings suggest that when employing the dual impeller, the micromixing time is reduced compared to a single impeller, but in the case of a high viscosity solution, the dual impeller does not shorten the micromixing time. At the same time, there is a decrease in energy dissipation as the reactor sizes increase.

The CFD modeling carried out to assess the flow profile and strain rates of the viscous solutions at various scales and at a constant tip speed of 1.83 m/s showed that for viscous solutions, at small scales, turbulent forces dominate the flow profile. However, as the reactor scale increases, the viscous forces significantly affect the flow. This is evident in the higher strain rates experienced by the least viscous solutions at small scales, and this condition shifts to the more viscous solutions as the scale increases. Moreover, while higher strain rates are observed at the impeller at small scales, it becomes concentrated at the baffles and reactor wall as the scale increases. This can be observed from the results obtained where the strain rates for the 0.1 L crystallizer differ greatly from the results for the 1 and 100 m^3^ crystallizers. For these reasons, it is indeed vital to analyze and consider the viscosity of the solutions during scale up, as it could significantly affect the final product quality as well as the optimization of the process.

## Supporting Information Available

Apparent viscosity measurements, solubility data reported in the literature and xylose solubility measured in the present work, reactor specifications, data used in calculations of the minimum agitation speed, results of scaling up based on impeller tip speed for xylitol, results of scaling up based on the average value of energy dissipation for xylitol, results of scaling up based on impeller tip speed for xylose, results of scaling up based on the average value of energy dissipation for xylose, results of scaling up based on impeller tip speed for glucose, results of scaling up based on the average value of energy dissipation for glucose, data used in calculations of xylitol mass transfer coefficient in a 100 m^3^ crystallizer, strain rates of xylitol (left) and erythritol (right) (40 °C) at 1 m^3^ scale dual impeller at 1.83 m/s tip speed, and velocity profiles (PDF)

## Abbreviations

CFD: computational fluid dynamics
CR: cooling rate
CRY-ML suspension: crystal-mother liquor suspension
HPAEC: high-performance anion exchange chromatography
ML: initial crystal-free mother liquor
*m*_obt_: obtained crystal mass
MRF: multiple reference frame
*m*_th_: theoretical crystal mass
PSD: particle size distribution
SEM: scanning electron microscopy
