# Supplementary material for: Influence of Viscosity on Variously Scaled Batch Cooling Crystallization from Aqueous Erythritol, Glucose, Xylitol, and Xylose Solutions
Source: Cryst Growth Des. 2024 Mar 21;24(7):2700–12. doi: 10.1021/acs.cgd.3c01136 (PMC10995941; doi:10.1021/acs.cgd.3c01136)
Supplement: Supplementary file 1 — cg3c01136_si_001.pdf [file cg3c01136_si_001.pdf]

Influence of viscosity on variously scaled batch cooling crystallization from aqueous erythritol, glucose, xylitol, and xylose solutions

Anna Zaykovskaya [a,†], *Bernadeth Amano, Marjatta Louhi-Kultanen* [a,†*]

[a]Department of Chemical and Metallurgical Engineering, School of Chemical Engineering, Aalto University, 02150, Espoo, Finland

[†]These authors contributed equally to this work

[*]Email: marjatta.louhi-kultanen@aalto.fi

Table A.1. Apparent viscosity measurements

Studied material	Viscosity, 10^{-3} Pa s	T, °C	rpm	Shear rate, s^{-1}
Xylitol	1092	20	0.3	0.064
	693	25		
	449	30		
	336	35	4	0.848
	90.42	40		
Erythritol	3.5	20	20	4.24
	3	25		
	2.6	30	50	10.6
	2.3	35		
	3.394	40	20	4.24
Glucose	1040	20	0.3	0.064
	703	25	0.5	0.106
	546	30	1	0.212
	374	35		
	14	40	30	6.36
Xylose	140	20	2	0.424
	64	25	6	1.272
	43	30	10	2.12
	29	35	20	4.24
	13.68	40	30	6.36

Table A.2. Solubility data reported in the literature and xylose solubility measured in the present work.

T °C	Solubility g/100 g of water				
	Erythritol [17]	Glucose [19]	Xylose (present work)	Xylose [15,16]	Xylitol [18]
20	47	90	118	117	170.3
25	57	-	130.9	131.48	183.3
30	67	120	-	-	233.3
40	89	160	174.3	-	300
50	122	-	216.5	-	400
60	156	290	-	-	170.3

Table A.3. Reactor specifications

INITIAL DATA			
TANK	TANK WITH ELLIPTICAL BOTTOM		
Inside diameter	1020	4734	mm
Total tank height	1310	6070	mm
Total volume	1	100	m^3
Level of media	1060	4940	mm
Volume of media	800	80000	L
BAFFLE	FLAT BAFFLE-1		
Number	4	4	
Width	100	464	mm
Length	1100	5110	mm
Dist. from bottom	200	928	mm
Angle to radius (fi)	0	0	Deg
IMPELLER	PITCHED BLADE.DUAL		
Tip diameter (D)	350	1620	mm
Number of impellers	1 or 2	1 or 2	
Dist. between stages	400	1860	mm
Pitch angle	45	45	Deg
Number of blades	6	6	
Width of blade	100	464	mm
Dist. from bottom	200	928	mm
Rotational speed	50	36	Rpm
Motor power	3	34.3	kW

Table A.4. Data used in calculations of the minimum agitation speed. *S* geometrical constant (dimensionless); ρ_L liquid density (kg/m^3); ρ_s solid density (kg/m^3); $\Delta\rho$ density difference between solid and liquid (kg/m^3); η dynamic viscosity (10^{-3} Pa·s); ν kinematic viscosity (m^2/s); X solid concentration in liquid (%) D_A diameter of agitator (mm); g acceleration due to gravity (m/s^2); L particle diameter (μm); N_{js} just suspension speed (rps/rpm) ; T = 20 °C

100 mL reactor

Material	S*	ρ_L, kg/m^3	ρ_s, kg/m^3	$\Delta\rho$, kg/m^3	η, 10^{-3} Pa·s	ν, m^2/s	L, μm		D_A, mm	g, m/s^2	N_{js}, rps	N_{js}, rpm
Erythritol	4.6	1078	1450	372	2.95	2.73E-03	423	X, %	38	9.81	5.76	345.55
Xylitol		1244	1520	276	904	7.27E-01	480				8.80	527.96
Glucose		1197	1560	363	1040	8.69E-01	200				8.45	507.24
Xylose		1233	1520	287	140	1.14E-01	400				6.77	406.31

1 m^3 and 100 m^3 reactors

Material	S[27]	ρ_L, kg/m^3	ρ_s, kg/m^3	$\Delta\rho$, kg/m^3	η, 10^{-3} Pa·s	ν, m^2/s	L, μm	X, %	D_A, mm 1 m^3 reactor	D_A, mm 100 m^3 reactor	g, m/s^2	N_{js}, rps 1 m^3	N_{js}, rpm 1 m^3	N_{js}, rps 100 m^3	N_{js}, rpm 100 m^3
Erythritol	4.6	1078	1450	372	2.95	2.73E-03	423	25.64	350	1620	9.81	0.87	52.34	0.24	14.23
Xylitol		1244	1520	276	904	7.27E-01	480	34.63	350	1620		1.33	79.98	0.36	21.74
Glucose		1197	1560	363	1040	8.69E-01	200	28.92	350	1620		1.28	76.84	0.35	20.89
Xylose		1233	1520	287	140	1.14E-01	400	21.60	350	1620		1.03	61.55	0.28	16.73

* Mak, A. Solid-liquid mixing in mechanically agitated vessels, Ph.D. Dissertation, University of London, London, U.K., 1992. https://discovery.ucl.ac.uk/id/eprint/1317906/1/299136.pdf

Table A.5. Results of scaling up based on impeller tip speed for xylitol.

Temperature	40 °C		20 °C	
Tank volume, m^3	1	100	1	100
Tank diameter, mm	1020	4734	1020	4734
Rotation speed, rpm	100	21.6	100	21.6
**Impeller TIP speed, m/s**	**1.83**	**1.80**	**1.84**	**1.80**
Energy dissipation – average value, W/kg<br>Single/Dual impeller	0.105/0.190	0.0230/0.041	0.106/0.191	0.0230/0.041
Maximum local energy dissipation rate, W/kg<br>Single/Dual impeller	8.76/7.67	1.90/1.70	8.78/7.7	1.90/1.7
Turbulent shear rate near the impeller blades, 1/s	354/332	160/150	581/544	270/250
Micromixing, s<br>Single/Dual impeller	216/220	470/470	62.9/56.3	140/120
Impeller Reynolds number	2910	13000	7790	36000

Table A.6. Results of scaling up based on the average value of energy dissipation for xylitol.

Temperature	40 °C		20 °C	
Tank volume, m^3	1	100	1	100
Tank diameter, mm	1020	4734	1020	4734
Rotation speed, rpm	100	36	100	36.0
Impeller TIP speed, m/s	1.83	3.10	1.84	3.10
**Energy dissipation – average value, W/kg** Single/Dual impeller	**0.106/0.190**	**0.110/0.190**	**0.106/0.191**	**0.110/0.190**
Maximum local energy dissipation rate, W/kg Single/Dual impeller	8.76/7.67	8.80/7.70	8.78/7.7	8.80/7.7
Turbulent shear rate near the impeller blades, 1/s Single/Dual impeller	354/332	350/330	581/544	580/540
Micromixing, s Single/Dual impeller	216/220	220/220	62.9/56.3	63/56
Impeller Reynolds number	2910	22000	7790	60000

Table A.7. Results of scaling up based on impeller TIP speed for xylose.

Temperature	40 °C		20 °C	
Tank volume, m^3	1	100	1	100
Tank diameter, mm	1020	4734	1020	4734
Rotation speed, rpm	100	21.6	100	21.6
**Impeller TIP speed, m/s**	**1.83**	**1.80**	**1.84**	**1.80**
Energy dissipation – average value, W/kg Single/Dual impeller	0.106/0.189	0.0230/0.041	0.106/0.191	0.0230/0.041
Maximum local energy dissipation rate, W/kg Single/Dual impeller	8.72/7.64	1.90/1.60	8.77/7.69	1.90/1.70
Turbulent shear rate near the impeller blades, 1/s	904/846	420/390	809/757	380/350
Micromixing, s Single/Dual impeller	29.5/20.05	63/44	34.3/25.7	74/55
Impeller Reynolds number	19000	88000	15200	70000

Table A.8. Results of scaling up based on the average value of energy dissipation for xylose.

Temperature	40 °C		20 °C	
Tank volume, m^3	1	100	1	100
Tank diameter, mm	1020	4734	1020	4734
Rotation speed, rpm	100	36	100	36.0
Impeller TIP speed, m/s	1.83	3.10	1.84	3.10
**Energy dissipation – average value, W/kg** Single/Dual impeller	**0.106/0.189**	**0.110/0.190**	**0.106/0.191**	**0.110/0.190**
Maximum local energy dissipation rate, W/kg Single/Dual impeller	8.72/7.64	8.70/7.60	8.77/7.69	8.80/7.70
Turbulent shear rate near the impeller blades, 1/s Single/Dual impeller	904/846	900/850	809/757	810/760
Micromixing, s Single/Dual impeller	29.5/20.5	29/21	34.3/25.7	34/26
Impeller Reynolds number	19000	1500000	15200	1200000

Table A.9. Results of scaling up based on impeller TIP speed for glucose.

Temperature	40 °C		20 °C	
Tank volume, m^3	1	100	1	100
Tank diameter, mm	1020	4734	1020	4734
Rotation speed, rpm	100	21.6	100	21.6
**Impeller TIP speed, m/s**	**1.83**	**1.80**	**1.84**	**1.80**
Energy dissipation – average value, W/kg Single/Dual impeller	0.106/0.189	0.0230/0.040	0.106/0.190	0.0230/0.041
Maximum local energy dissipation rate, W/kg Single/Dual impeller	8.72/7.64	1.90/1.60	8.77/7.68	1.90/1.70
Turbulent shear rate near the impeller blades, 1/s	913/855	420/400	1060/988	490/460
Micromixing, s Single/Dual impeller	29.1/20.1	63/43	25/15.8	54/34
Impeller Reynolds number	19400	90000	25800	120000

Table A.10. Results of scaling up based on the average value of energy dissipation for glucose.

Temperature	40 °C		20 °C	
Tank volume, m^3	1	100	1	100
Tank diameter, mm	1020	4734	1020	4734
Rotation speed, rpm	100	36	100	36.0
Impeller TIP speed, m/s	1.83	3.10	1.84	3.10
**Energy dissipation – average value, W/kg** Single/Dual impeller	**0.106/0.189**	**0.110/0.190**	**0.106/0.190**	**0.110/0.190**
Maximum local energy dissipation rate, W/kg Single/Dual impeller	8.72/7.64	8.70/7.60	8.77/7.68	8.80/7.70
Turbulent shear rate near the impeller blades, 1/s Single/Dual impeller	913/855	910/850	1060/988	1100/990
Micromixing, s Single/Dual impeller	29.1/20.1	29/20	25/15.8	25/16
Impeller Reynolds number	19400	150000	25800	200000

The expression reported by Levins and Glastonbury for calculating mass transfer coefficient is shown below:

$$Sh = 2 + 0.47\left(\frac{\varepsilon L^4}{\nu^3}\right)^{0.62} Sc^{0.36}\left(\frac{D_A}{D_T}\right)^{0.17} \quad \text{(A.1)}$$

where *Sh*	Sherwood number (k_L L/D),	Dimensionless
ε	power input per unit mass of fluid,	m^2/s^3
L	particle diameter,	m
ν	kinematic viscosity,	m^2/s
Sc	Schmidt number (ν/D),	Dimensionless
D_A	diameter of agitator	m
D_T	diameter of tank	m
k_L	mass transfer coefficient,	m/s
D_{mol}	molecular diffusivity,	m^2/s

Table A.11. Data used in calculations of xylitol mass transfer coefficient in a 100 m^3 crystallizer. The particle size used in calculations was 480 μm.

T, °C	ρ, kg/m^3	μ, Pa s	ν, m^2/s	Re_{mixer}	N_p	P, W	ε, W/kg	D_{mol}, m^2/s*	D_A, m	D_T, m	Sh	k_L, m/s
20	1249	0.0365	2.92×10^{-5}	53897.65	6.26	18843.67	0.183019	7.58×10^{-10}	1.6	4.7	11.78	1.9×10^{-5}
40	1287	0.0905	7.03×10^{-5}	22400.37	6.23	19323.93	0.187684	1.06×10^{-9}	1.6	4.7	4.36	9.6×10^{-6}

*Winkelmann, J. *Diffusion in Gases, Liquids and Electrolytes*; 2018. https://doi.org/10.1007/978-3-662-54089-3.

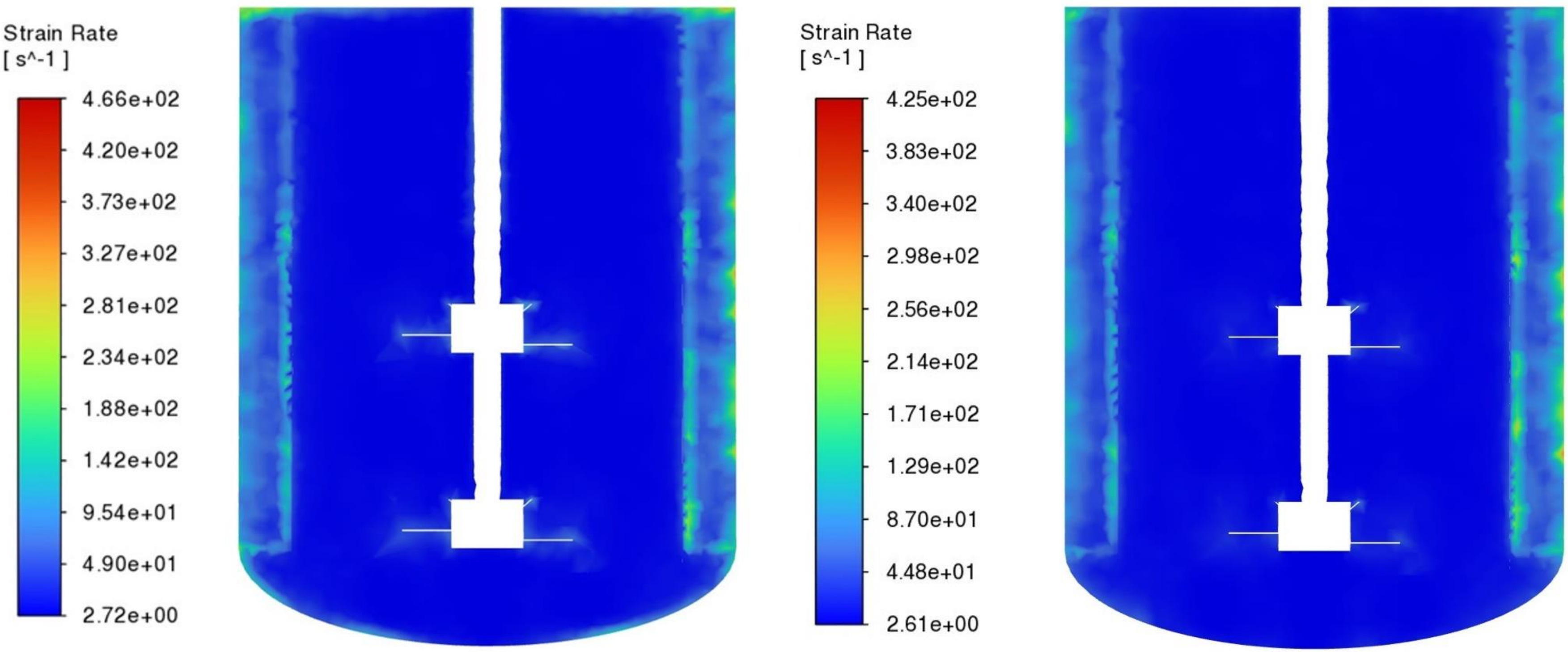

Figure A.1. Strain rates of xylitol (left) and erythritol (right) (40 °C) at 1 m^3-scale dual pitched blade at 1.83 m/s tip speed.

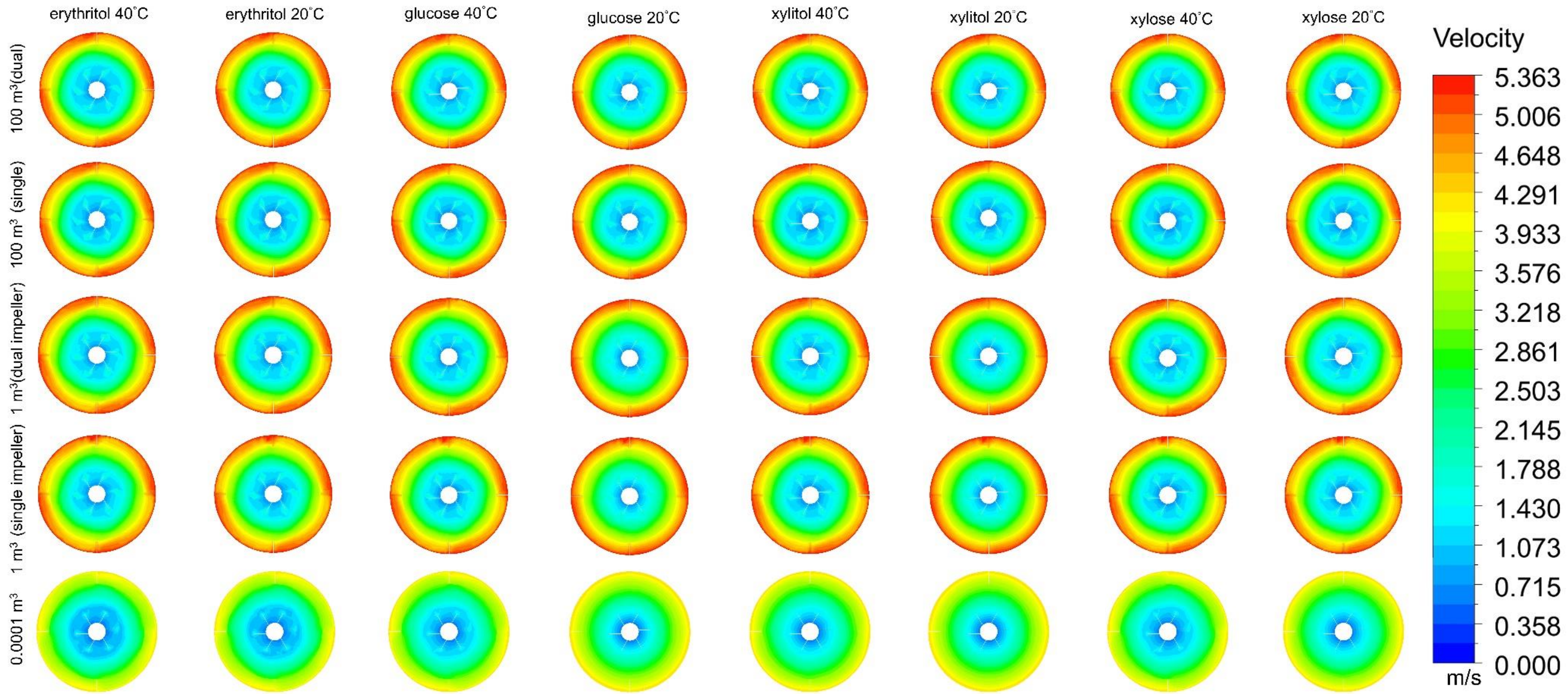

Figure A.2. Velocity profiles.

Mixing time measurements in 1 liter crystallizer

Additional VisiMix simulations and experiments with a 1-liter crystallizer were carried out to investigate the influence of viscosity on mixing times in saturated xylitol solutions at 20 and 40 °C. One liter is the smallest reactor volume suitable for VisiMix simulations. The obtained responses are presented in Figure A.3. In these experiments in terms of macromixing, the same quantity of acid during some seconds was added batchwise to the equal volume of saturated xylitol solution. The tip speed was 1.28 m/s. The change in pH was monitored using a pH meter and video camera recording. The pH response curves, as illustrated in the resulting plot, clearly show that the mixing time increases significantly as the viscosity of the solution increases. The obtained mixing time results to VisiMix simulation results on the time for unmixed part of media to reach 1 % were compared. The results show that both simulations and laboratory experiments demonstrate a consistent trend within a comparable timeframe.

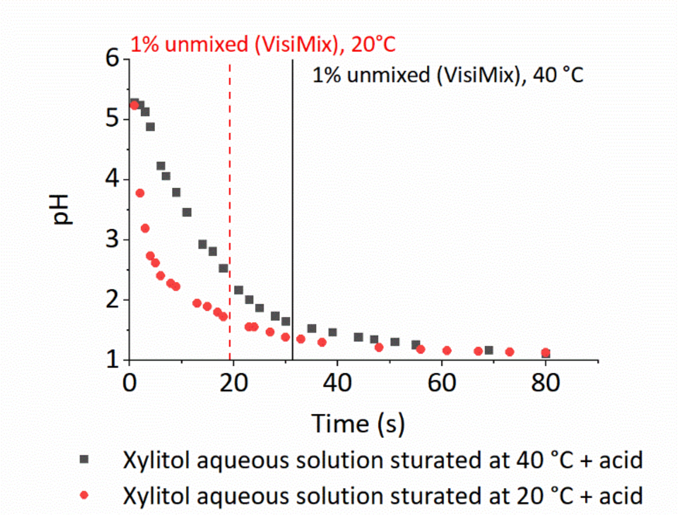

Figure A.3. Mixing time experiment vs simulation
